# Dataset on insight into inhibition capacity of cyclic tetra-amino acid based compounds as efficient crystal structure of human insulin-degrading enzyme inhibitors

**DOI:** 10.1016/j.dib.2025.111944

**Published:** 2025-08-05

**Authors:** Cecilia O. Akintayo, Abel Kolawole Oyebamiji, Arogundade Ibukun O, Adeniyi Adebayo, Sunday Adewale Akintelu, Samson Olusegun Afolabi, Oluwakemi Ebenezer

**Affiliations:** a Chemistry Department, Federal University Oye Ekiti, Ekiti State, Nigeria; b Department of Industrial Chemistry, University of Ilesa, Ilesa, Osun State, Nigeria; c Good Health and Wellbeing Research Clusters (SDG 03), University of Ilesa, Ilesa, Osun State, Nigeria; d Department of Chemistry, Federal University of Agriculture, Abeokuta, Ogun State, Nigeria

**Keywords:** Crystal, diabetes, Insulin, Peptides, Simulation

## Abstract

The biochemical evaluation of nine cyclopeptide derivatives were investigated using computational approaches. Key software in drug design and discovery (Spartan for optimization; Molecular operating environment (MOE) for induced fit docking; ADMETSar for pharmacokinetics; GROMACS for molecular dynamic simulation) were employed in this work. The optimization was accomplished using 6–31G* as basis set via Spartan 14 software while the optimized compounds were docked against human insulin-degrading enzyme (2WBY). The embedded features of the optimized compounds were accessible after optimization and they were observed and reported. Also, methyl (3S,9S,12S)-12-(1,3-dioxoisoindolin-2-yl)-9-(4-hydroxybenzyl)-5,8,11-trioxo-4,7,10-triaza-1(1,3)-benzenacyclotridecaphane-3-carboxylate (compound **1**) proved to possess greater potential to inhibit human insulin-degrading enzyme (2WBY) than other examined compounds and the reference compound (Metformin). The pharmacokinetic evaluation of compound with highest binding affinity and reference compound were executed and reported. Also, other activity of the lead compound were explored using SwissTargetPrediction software and the outcome were observed and reported

Specifications Table

**Table:** 

Subject	*Chemoinformatics*
Specific subject area	Drug Design and Evaluation
Type of data	Figure, Chart, Table, ADMET, Graph
Data collection	The designed peptides were optimized at B3LYP via 6–31* in three phases (vacuum, water and Ethanol) using Spartan 14. The electronic properties of individual compounds were accessible after optimization and were subjected to further investigation. The human insulin-degrading enzyme (2WBY) was docked against the entire compounds using molecular operating environment software (version 2024.06). The lead compound and the reference compound were subjected to Gromacs software for molecular dynamic simulation so as to calculate the actual binding energy, root mean square deviation (RMSD), root mean square fluctuation (RMSF), radius of gyration (ROG) and hydrogen bond (HB). pharmacokinetics evaluation was exhibited on compound with highest binding affinity. Also, further investigation were explored to identify other area of bioactivity of the lead compound and the outcomes were observed and reported.
Data source location	Computational Chemistry Research Laboratory, Department of Industrial Chemistry, University of Ilesa, Ilesa, Osun State, Nigeria.
Data accessibility	https://data.mendeley.com/datasets/7876zz7vtg/1 Repository name: Mendeley Data Data identification number: 10.17632/7876zz7vtg.1 Direct URL to data: https://data.mendeley.com/datasets/7876zz7vtg/1 Instructions for accessing these data: The data can be accessed using the above URL
Related research article	*None*

## 1 Value of the Data

• The calculated descriptors will show to scientists the effect of the derivatives on the geometries of the compounds under investigation.
• The descriptors for all the molecules used in this study will help researchers determine the types of reactions the examined compound may undergo.
• The calculated binding affinity and binding energy obtained from the examined complexes will show to the researchers the extent at which the compounds binds with the receptors.
• The number and the type of amino acid residues that are involved in the interaction will be exposed to the scientists.
• The pharmacokinetic analysis results will offer insights into the absorption, digestion, metabolism, and excretion processes of the molecule with the greatest binding affinity in the human body.
• The extended biological activities of the lead compound will expose researchers to identify various bioactivities that could be carried out with the lead compound.

## 2 Background

The objectives of this work are:

➢ To examine the influence of electron-withdrawing and electron-donating groups on the properties of the parent compounds.
➢ To investigate the effect of solvent on the bond joining atoms together as well as the geometries of the examined parent compound.
➢ To investigate the biological activities of the compounds under investigation against the human insulin-degrading enzyme (2WBY) through molecular modeling techniques.
➢ To assess the potential of the compound with the highest binding affinity to function as a therapeutic agent through pharmacokinetic evaluation.

## 3 Data Description

Table 1 presents the 2D and 3D structures of the compound under investigation, along with its IUPAC name. As depicted in Table 1, the 3D structure of the compound consists of atoms represented by color-coded spheres (red for oxygen, blue for nitrogen, and yellow for sulfur), which are bonded together to form the molecular configuration of the compound being analyzed.

**Table 1:** 2-Dimensional and 3-dimensional Structures and IUPAC name of the Investigated compounds.

Table 2 exposed the calculated descriptors generated from molecules optimized in vacuum, water and ethanol. The selected descriptors for the optimized molecules were highest occupied molecular orbital energy (EHOMO), lowest unoccupied molecular orbital energy (ELUMO) and energy gap.

**Table 2:** Calculated descriptors from molecules in vacuum, water and Ethanol.

	Vacuum			Water			Ethanol		
	HOMO (eV)	LUMO (eV)	Energy Gap (eV)	HOMO (eV)	LUMO (eV)	Energy Gap (eV)	HOMO (eV)	LUMO (eV)	Energy Gap (eV)
**1**	−5.74	−1.97	3.77	−5.91	−2.19	3.72	−5.74	−1.97	3.77
**2**	−6.45	−2.24	4.21	−6.49	−2.26	4.23	−6.45	−2.24	4.21
**3**	−6.16	−2.48	3.68	−6.48	−2.2	4.28	−6.16	−2.48	3.68
**4**	−6.16	−2.4	3.76	−6.49	−2.16	4.33	−6.16	−2.4	3.76
**5**	−5.56	−2.57	2.99	−5.4	−2.27	3.13	−5.56	−2.57	2.99
**6**	−6.51	−2.22	4.29	−6.5	−2.25	4.25	−6.51	−2.22	4.29
**7**	−5.99	−2.4	3.59	−6.36	−2.18	4.18	−5.99	−2.4	3.59
**8**	−6.26	−2.05	4.21	−6.5	−2.28	4.22	−6.26	−2.05	4.21
**9**	−6.17	−1.99	4.18	−6.17	−1.99	4.18	−6.17	−1.99	4.18

**Fig. 1:**
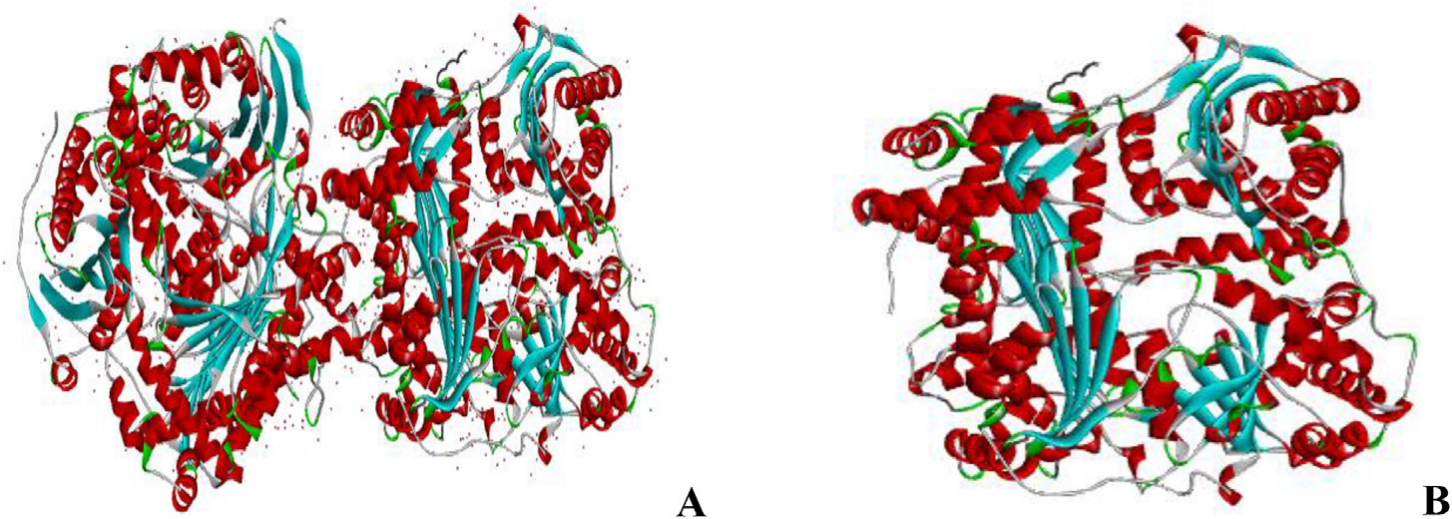
Unclean (A) and clean receptor (B).

Fig. 1 showed the downloaded unclean and clean human insulin-degrading enzyme (2WBY). The unclean receptor comprised of water molecules, small molecules and the investigated enzyme. And Table 3 showed the calculated binding affinity for compound 1–9 in kcal/mol. The calculated binding affinity for compound 1–9 docked against human insulin-degrading enzyme (2WBY) were −9.4382kcal/mol, −7.1238 kcal/mol, −8.1002 kcal/mol,−8.3966 kcal/mol, −8.5956 kcal/mol,−8.1910 kcal/mol, −7.7368 kcal/mol,−7.6872 kcal/mol, −7.0595 kcal/mol and −5.0000 kcal/mol (Metformin) .

**Table 3:** Calculated scoring for compound 1–9 in kcal/mol.

	Scoring (kcal/mol)
1	−9.4382
2	−7.1238
3	−8.1002
4	−8.3966
5	−8.5956
6	−8.1910
7	−7.7368
8	−7.6872
9	−7.0595
Ref	−5.0000

Ref: Metformin.

Table 4 revealed the non-bonding interaction and visual representation for the investigated compounds and human insulin-degrading enzyme (2WBY). The calculated distance, bond type, residue observed between individual compound and the receptor was (2.70, 3.07, 2.94, 3.36); (H-donor, H-acceptor, H- acceptor, H-acceptor); (ASN 139, SER 138, ASN 139, ARG 824); for compound **1**; (3.31, 3.61, 4.50, 3.82, 3.73); (H-donor, H-pi, pi-H, pi-H, pi-pi); (SER 138, PHE 115, SER 138, SER 138, PHE 820) for compound **2**; (2.84, 3.33, 3.03, 3.42, 3.55); (H-donor, H-acceptor, H-acceptor, H-acceptor, pi-pi); (ASN 139, ARG 824, ARG 824, VAL 833, PHE 820) for compound **3**; (4.28, 4.07, 3.76); (H-pi, pi-H, pi-pi); (PHE 820, SER 138, PHE 820) for compound **4**; (2.91, 3.91); (H-acceptor, H-pi); (ASN 139, HIS 112) for compound **5**; (3.28); (H-acceptor); (ASN 139) for compound **6**; (3.01, 2.98); (H-acceptor, H-acceptor); (SER 128, SER 128) for compound **7**; (3.52); (pi-pi); (PHE 820) for compound **8**; (3.12, 3.05, 3.62); (H-donor, H-acceptor, pi-H); (GLU 809, GLY 842, ARG 920) for compound **9**.

**Table 4:** Predicted non-bonding interaction and visual representation for the investigated complexes.

Table 5, Table 6 present the pharmacokinetic analysis of the lead compound (compound 1) and Metformin (reference compound). The analysis is provided in two formats: ADMET predicted profile — classification and ADMET predicted profile — regression. The prediction outcomes are categorized under three main headings: model, result, and probability. For absorption, the predicted models include Blood-Brain Barrier permeability, Human Intestinal Absorption, Caco-2 Permeability, P-glycoprotein Substrate, P-glycoprotein Inhibitor, and Renal Organic Cation Transporter. For distribution, the model considered is Subcellular Localization. The metabolism section includes predictions for CYP450 2C9, CYP450 2D6, CYP450 3A4 Substrates, along with inhibitors for CYP450 1A2, CYP450 2C9, CYP450 2D6, CYP450 2C19, CYP450 3A4, and CYP Inhibitory Promiscuity. Toxicity predictions include Human Ether-a-go-go-Related Gene Inhibition, AMES Toxicity, Carcinogenicity, Fish Toxicity, Tetrahymena Pyriformis Toxicity, Honey Bee Toxicity, Biodegradation, Acute Oral Toxicity, and Carcinogenicity (Three-class). Additionally, for the ADMET predicted profile — regression, the factors considered include Aqueous Solubility, Caco-2 Permeability (absorption), as well as Rat Acute Toxicity, Fish Toxicity, and Tetrahymena Pyriformis Toxicity (toxicity).

**Table 5:** ADMET predicted profile — classification for compound 1.

Model	Result	Probability
**Absorption**		
Blood-Brain Barrier	BBB-	0.7435
Human Intestinal Absorption	HIA+	0.8920
Caco-2 Permeability	Caco2-	0.7514
P-glycoprotein Substrate	Substrate	0.7572
P-glycoprotein Inhibitor	Non-inhibitor	0.9140
	Non-inhibitor	0.7035
Renal Organic Cation Transporter	Non-inhibitor	0.8595
**Distribution**		
Subcellular localization	Mitochondria	0.5829
**Metabolism**		
CYP450 2C9 Substrate	Non-substrate	0.7819
CYP450 2D6 Substrate	Non-substrate	0.8448
CYP450 3A4 Substrate	Substrate	0.5866
CYP450 1A2 Inhibitor	Non-inhibitor	0.9422
CYP450 2C9 Inhibitor	Inhibitor	0.5597
CYP450 2D6 Inhibitor	Non-inhibitor	0.8387
CYP450 2C19 Inhibitor	Non-inhibitor	0.6628
CYP450 3A4 Inhibitor	Non-inhibitor	0.6824
CYP Inhibitory Promiscuity	Low CYP Inhibitory Promiscuity	0.7665
**Excretion**		
**Toxicity**		
Human Ether-a-go-go-Related Gene Inhibition	Weak inhibitor	0.9760
	Non-inhibitor	0.5524
AMES Toxicity	Non AMES toxic	0.8325
Carcinogens	Non-carcinogens	0.8848
Fish Toxicity	High FHMT	0.9683
Tetrahymena Pyriformis Toxicity	High TPT	0.9501
Honey Bee Toxicity	Low HBT	0.7811
Biodegradation	Not ready biodegradable	0.9608
Acute Oral Toxicity	III	0.6142
Carcinogenicity (Three-class)	Non-required	0.6199
**ADMET Predicted Profile — Regression**		
**Model**	**Value**	**Unit**
**Absorption**		
Aqueous solubility	−2.8902	LogS
Caco-2 Permeability	−0.0692	LogPapp, cm/s
**Toxicity**		
Rat Acute Toxicity	2.5853	LD50, mol/kg
Fish Toxicity	1.1939	pLC50, mg/L
Tetrahymena Pyriformis Toxicity	0.3888	pIGC50, ug/L

**Table 6:** ADMET predicted profile — classification for metformin.

Model	Result	Probability
**Absorption**		
Blood-Brain Barrier	BBB+	0.5868
Human Intestinal Absorption	HIA+	0.9156
Caco-2 Permeability	Caco2-	0.8958
P-glycoprotein Substrate	Non-substrate	0.6643
P-glycoprotein Inhibitor	Non-inhibitor	0.9613
	Non-inhibitor	0.8892
Renal Organic Cation Transporter	Non-inhibitor	0.7518
**Distribution**		
Subcellular localization	Lysosome	0.7916
**Metabolism**		
CYP450 2C9 Substrate	Non-substrate	0.7929
CYP450 2D6 Substrate	Non-substrate	0.7325
CYP450 3A4 Substrate	Non-substrate	0.6906
CYP450 1A2 Inhibitor	Non-inhibitor	0.9046
CYP450 2C9 Inhibitor	Non-inhibitor	0.9159
CYP450 2D6 Inhibitor	Non-inhibitor	0.9231
CYP450 2C19 Inhibitor	Non-inhibitor	0.9130
CYP450 3A4 Inhibitor	Non-inhibitor	0.9506
CYP Inhibitory Promiscuity	Low CYP Inhibitory Promiscuity	0.9763
**Excretion**		
**Toxicity**		
Human Ether-a-go-go-Related Gene Inhibition	Weak inhibitor	0.9807
	Non-inhibitor	0.9274
AMES Toxicity	Non AMES toxic	0.7367
Carcinogens	Non-carcinogens	0.6691
Fish Toxicity	Low FHMT	0.8761
Tetrahymena Pyriformis Toxicity	Low TPT	0.9005
Honey Bee Toxicity	Low HBT	0.5816
Biodegradation	Not ready biodegradable	0.9380
Acute Oral Toxicity	III	0.7817
Carcinogenicity (Three-class)	Non-required	0.6357
**ADMET Predicted Profile — Regression**		
**Model**	**Value**	**Unit**
**Absorption**		
Aqueous solubility	−0.9180	LogS
Caco-2 Permeability	0.2041	LogPapp, cm/s
**Toxicity**		
Rat Acute Toxicity	1.7407	LD50, mol/kg
Fish Toxicity	2.2781	pLC50, mg/L
Tetrahymena Pyriformis Toxicity	−0.6614	pIGC50, ug/L

**Fig. 2:**
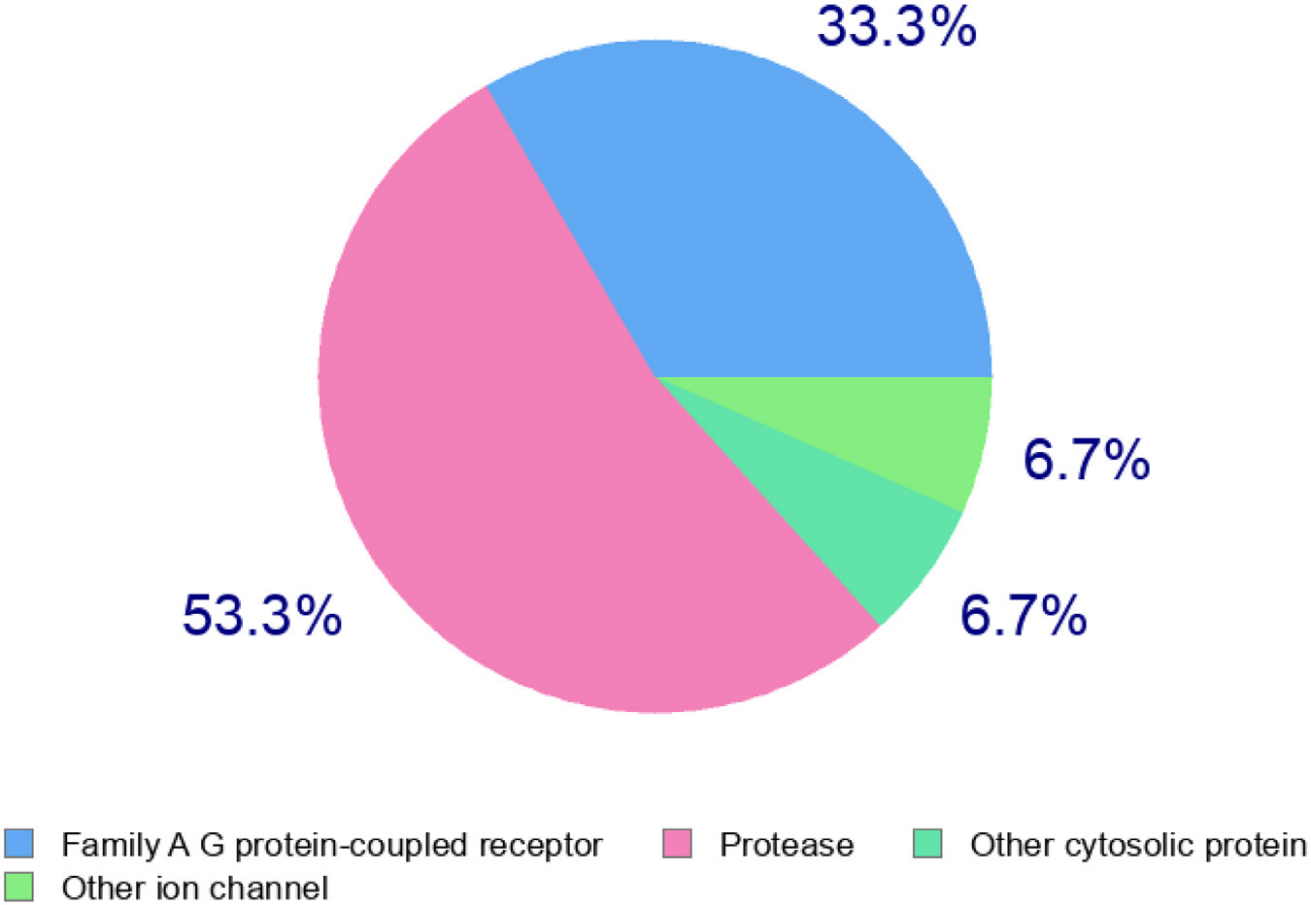
Pictorial representation of other bio-targets and their probabilities for compound 1.

Table 7 and Fig. 2 showed other biological targets, common names, target class, probability and Known actives (3D/2D) that compound 1 (Lead compound) has potential ability to inhibit. The name of the targets are Mu opioid receptor (by homology), Delta opioid receptor (by homology), Matrix metalloproteinase 9, Matrix metalloproteinase 1, Matrix metalloproteinase 3, Renin, Matrix metalloproteinase 2, Kappa Opioid receptor, Cystinyl aminopeptidase, Aminopeptidase N, Type-1 angiotensin II receptor, Inhibitor of apoptosis protein 3, C—C chemokine receptor type 3, Cathepsin (B and K), Apoptosis regulator Bcl-2, Thrombin, Neurokinin 3 receptor, Cathepsin L, Acetyl-coenzyme A transporter 1, Pyruvate dehydrogenase kinase isoform 1, Integrin alpha-4/beta-1, Histone deacetylase 8, Histone deacetylase 1, Matrix metalloproteinase 8, Neurotensin receptor 1, Beta-secretase 1, Calpain 1, Cyclooxygenase-2, Methionyl-tRNA synthetase, Heat shock protein HSP 90-beta, Protein farnesyltransferase, Carbonic anhydrase II, Cystine/glutamate transporter, Neurokinin 2 receptor, Proenkephalin B, Cholecystokinin B receptor, Orexin receptor 2, Hepatocyte growth factor receptor, Gonadotropin-releasing hormone receptor, Tyrosine-protein kinase SRC, Voltage-gated N-type calcium channel alpha-1B subunit, G protein-coupled receptor kinase 5, c-Jun N-terminal kinase 3, Cathepsin K, Angiotensin-converting enzyme, Tyrosine-protein kinase HCK, LDL-associated phospholipase A2, MAP kinase p38 gamma, E3 SUMO-protein ligase CBX4.

**Table 7:** Other predicted biological targets and their probabilities for compound 1.

Target	Common name	Target Class	Probability*
Mu opioid receptor (by homology)	OPRM1	Family A G protein-coupled receptor	0.084065592
Delta opioid receptor (by homology)	OPRD1	Family A G protein-coupled receptor	0.084065592
Matrix metalloproteinase 9	MMP9	Protease	0.074564954
Matrix metalloproteinase 1	MMP1	Protease	0.074564954
Matrix metalloproteinase 3	MMP3	Protease	0.074564954
Renin	REN	Protease	0.074564954
Matrix metalloproteinase 2	MMP2	Protease	0.074564954
Kappa Opioid receptor	OPRK1	Family A G protein-coupled receptor	0.074564954
Cystinyl aminopeptidase	LNPEP	Protease	0.074564954
Aminopeptidase N	ANPEP	Protease	0.074564954
Type-1 angiotensin II receptor	AGTR1	Family A G protein-coupled receptor	0.074564954
Inhibitor of apoptosis protein 3	XIAP	Other cytosolic protein	0.074564954
C-C chemokine receptor type 3	CCR3	Family A G protein-coupled receptor	0.074564954
Cathepsin (B and K)	CTSB	Protease	0.074564954
Apoptosis regulator Bcl-2	BCL2	Other ion channel	0.074564954
Thrombin	F2	Protease	0.074564954
Neurokinin 3 receptor	TACR3	Family A G protein-coupled receptor	0.074564954
Cathepsin L	CTSL	Protease	0.074564954
Acetyl-coenzyme A transporter 1	SLC33A1	Electrochemical transporter	0.074564954
Pyruvate dehydrogenase kinase isoform 1	PDK1	Kinase	0.074564954
Integrin alpha-4/beta-1	ITGB1 ITGA4	Membrane receptor	0.074564954
Histone deacetylase 8	HDAC8	Eraser	0.074564954
Histone deacetylase 1	HDAC1	Eraser	0.074564954
Matrix metalloproteinase 8	MMP8	Protease	0.074564954
Neurotensin receptor 1	NTSR1	Family A G protein-coupled receptor	0.074564954
Beta-secretase 1	BACE1	Protease	0.074564954
Calpain 1	CAPN1	Protease	0.074564954
Cyclooxygenase-2	PTGS2	Oxidoreductase	0.074564954
Methionyl-tRNA synthetase	MARS	Enzyme	0.074564954
Heat shock protein HSP 90-beta	HSP90AB1	Other cytosolic protein	0.074564954
Protein farnesyltransferase	FNTA FNTB	Enzyme	0.074564954
Carbonic anhydrase II	CA2	Lyase	0.074564954
Cystine/glutamate transporter	SLC7A11	Electrochemical transporter	0.074564954
Neurokinin 2 receptor	TACR2	Family A G protein-coupled receptor	0.074564954
Proenkephalin B	PDYN	Other ion channel	0.074564954
Cholecystokinin B receptor	CCKBR	Family A G protein-coupled receptor	0.074564954
Orexin receptor 2	HCRTR2	Family A G protein-coupled receptor	0.074564954
Hepatocyte growth factor receptor	MET	Kinase	0.074564954
Gonadotropin-releasing hormone receptor	GNRHR	Family A G protein-coupled receptor	0.074564954
Tyrosine-protein kinase SRC	SRC	Kinase	0.074564954
Voltage-gated N-type calcium channel alpha-1B subunit	CACNA1B	Voltage-gated ion channel	0.074564954
G protein-coupled receptor kinase 5	GRK5	Kinase	0.074564954
c-Jun N-terminal kinase 3	MAPK10	Kinase	0.074564954
Cathepsin K	CTSK	Protease	0.074564954
Angiotensin-converting enzyme	ACE	Protease	0.074564954
Tyrosine-protein kinase HCK	HCK	Kinase	0.074564954
LDL-associated phospholipase A2	PLA2G7	Enzyme	0.074564954
MAP kinase p38 gamma	MAPK12	Kinase	0.074564954
E3 SUMO-protein ligase CBX4	CBX4	Enzyme	0.074564954

**Fig. 3:**
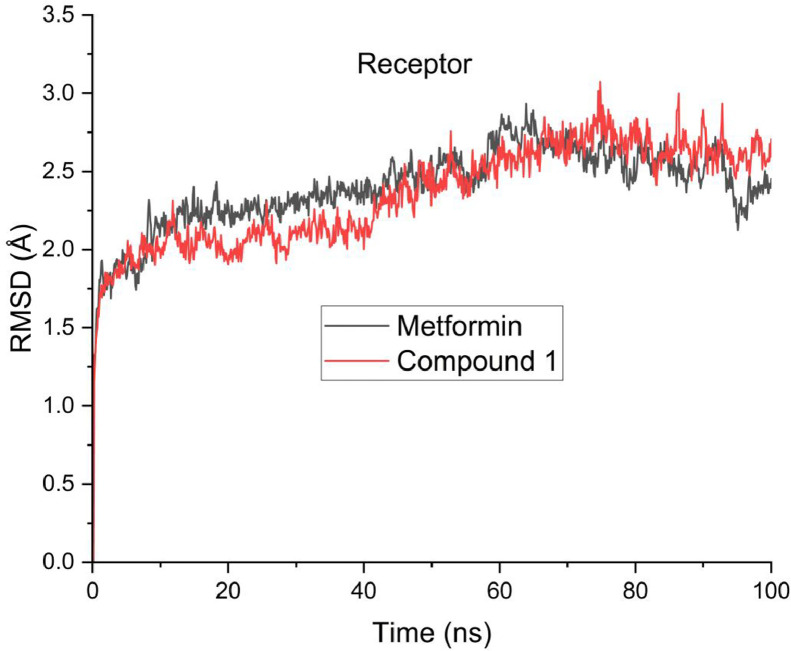
Predicted RMSD for compound 1 and Metformin.

**Fig. 4:**
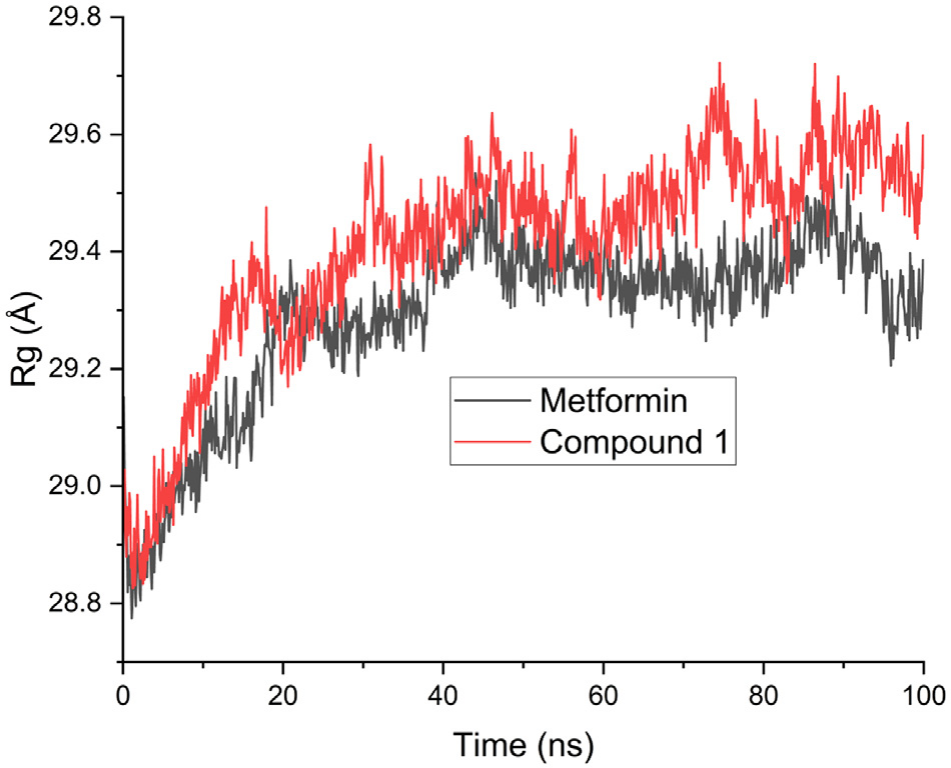
Predicted radius of gyration (ROG) for compound 1 and Metformin.

**Fig. 5:**
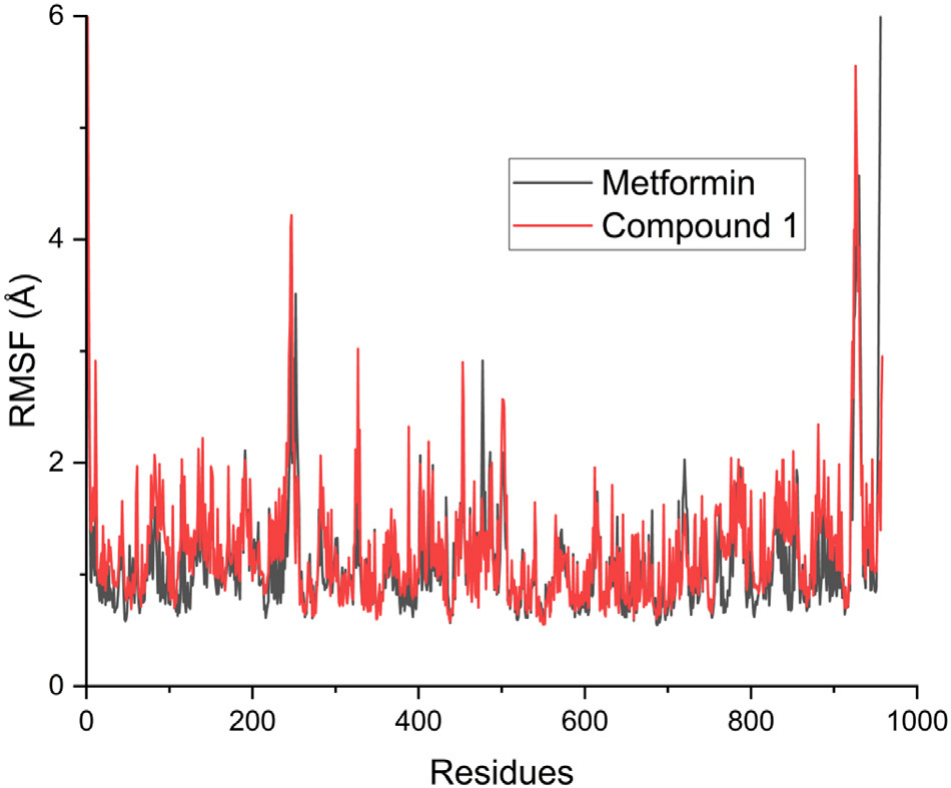
Predicted RMSF for compound 1 and Metformin.

Moreover, Table 8 revealed the calculated free binding energies for compound 1 and the reference compound. The calculated binding energy for compound 1 and the reference compound were −51.6717 and −17.2580 (E_VDWAALS_); −43.8724 and −36.3615 (**E_EL_**); 72.1903 and 34.8522 (**E_GB_**); −6.8590 and −2.9702 (**E_SURF_**); −95.5441 and −53.6195 (**∆G**_gas_); 65.3313 and 31.8820 (**∆G**_solv_); −30.2128 kcal/mol and −21.7375 kcal/mol (**∆G**). The predicted root mean square deviation (RMSD), radius of gyration (ROG) and root mean square fluctuation (RMSF) were displayed in Fig. 3, Fig. 4, Fig. 5 respectively.

**Table 8:** Calculated free energies for compound 1 and reference compound.

Energy term	Metformin	Compound 1
**E_VDWAALS_**	−17.2580	−51.6717
**E_EL_**	−36.3615	−43.8724
**E_GB_**	34.8522	72.1903
**E_SURF_**	−2.9702	−6.8590
**∆G**_gas_	−53.6195	−95.5441
**∆G**_solv_	31.8820	65.3313
**∆G (**kcal/mol**)**	−21.7375	−30.2128

## 4 Experimental Design, Materials and Methods

Nine cyclopeptides were modeled and optimized in a stepwise manner across three distinct phases: vacuum, water, and ethanol environments. These compounds were derivatives of a synthesized compound with efficient biological activities. These calculations were performed using Spartan '14 software [1,2]. Key electronic properties, including the Highest Occupied Molecular Orbital (HOMO), Lowest Unoccupied Molecular Orbital (LUMO), and the energy gap, were selected for each phase of the compounds under investigation. These descriptors were subsequently extracted and reported for each cyclopeptide.

For docking studies, the compounds were prepared using the Induced Fit Docking (IFD) method, which was implemented via the Molecular Operating Environment (MOE) software (version 2024.06). The target receptor, human insulin-degrading enzyme (PDB ID: 2WBY), was retrieved from the Protein Data Bank (PDB) [3]. Prior to docking, the receptor was processed to remove water molecules and any co-crystallized small molecules. The binding site for the receptor was identified using site finding tool and then saved in MOE-compatible format. More so, the ligands were minimized to ascertain the correct structural conformation and saved the ligands in .moe format. The ligand-receptor complexes were docked using the IFD method with 30 poses generated for each compound [4,5].

The docking calculations were conducted with the MOE software, with a focus on obtaining accurate binding modes and interactions. The docking calculation led to binding affinity for each ligand-receptor complex and the 2D and 3D for the docked complexes were examined to identify the type of nonbonding interaction involved in the investigated complexes. For the pharmacokinetic evaluation of compound 1 (the lead compound) and the reference compound, ADMETSar software [6,7] was employed to predict various ADMET (absorption, distribution, metabolism, excretion, and toxicity) properties. The results from these predictions were thoroughly analyzed, and the relevant data for both compounds were reported comprehensively. These evaluations provided insight into the potential pharmacokinetic behavior of the compounds in a biological system, aiding in their further development and optimization.

Furthermore, the docking results were validated through molecular dynamics (MD) simulations for both the reference drug and the potential hit candidate. The input files for the protein-ligand complexes were generated using the CHARMM-GUI platform (https://www.charmm-gui.org/), integrating the protein in PDB format along with the ligand in MOL2 format. A rectangular simulation box of 10 Å was created to facilitate accurate modeling of molecular interactions. The system was solubilized by incorporating water molecules and ions (0.15 M *K*+ and Cl-) to balance the charge. Files for parameters were produced using the AMBER force field (GAFF2), which is compatible with the AMBER ff19SB protein force field, as illustrated by Owolabi et al. [8]. The complexes underwent energy minimization for 100,000 steps, succeeded by a 5-nanosecond (ns) equilibration phase and a subsequent 100-ns production run. The equilibration took place under an NVT ensemble, whereas the production phase utilized an NPT ensemble, maintaining a consistent temperature of 310.15 K. Trajectory analysis was carried out using the CPPTRAJ module, and the binding interactions along with free energies (ΔGbind) of the protein-ligand complexes were evaluated using the Molecular Mechanics Generalized-Boltzmann Surface Area (MMGBSA) technique.

## Limitations

Further studies are warranted to deepen the understanding of the biological roles of the compounds investigated, extending beyond the scope of the current findings.

## Ethics Statement

This study does not involve studies with animals and humans.

## Data Availability

Mendeley DataDataset on Insight into Inhibition Capacity of Cyclic Tetra-Amino Acid based Compounds as Efficient Crystal structure of human insulin-degrading enzyme Inhibitors (Original data).
